# STAT3 activation in HER2-overexpressing breast cancer promotes epithelial-mesenchymal transition and cancer stem cell traits

**DOI:** 10.3892/ijo.2013.2195

**Published:** 2013-11-29

**Authors:** SEYUNG S. CHUNG, NOLAN GIEHL, YANYUAN WU, JAYDUTT V. VADGAMA

**Affiliations:** 1 Division of Cancer Research and Training, Charles R. Drew University of Medicine and Science;; 2 Department of Medicine, David Geffen School of Medicine, University of California, Los Angeles, CA, USA

**Keywords:** STAT3, breast cancer, ER, HER2, cancer stem cell, epithelial-mesenchymal transition

## Abstract

Clinically, HER2 proto-oncogene amplification is found in about 25–30% of human breast cancers, where it is correlated to a poor prognosis. Constitutive STAT3 activation is found in about 50–60% of the breast tumors and associated with tumorigenesis and drug resistance. In this study, we showed that STAT3 was phosphorylated in HER2-overexpressing, ER-positive human breast tumors and, furthermore, phosphorylated STAT3 promoted the stem-like cell phenotype. We examined the dysregulation of the stem cell markers (Oct-4, Sox-2 and CD44) and the tumorsphere formation in HER2-overexpressing human breast cancer cell lines. We demonstrated that the STAT3 inhibitor, Stattic, treatment abolished the cancer stem cell phenotype in HER2-positive breast cancers. Combined treatment of Herceptin and Stattic showed the synergistic effect on the cancer cell growth *in vitro*. In addition, when the STAT3 gene was knocked down, the expression of the stem cell markers Oct-4, Sox-2 and CD44 were downregulated and tumorsphere formation was abolished. HER2-elicited STAT3 signaling may provide a potential model for drug resistance induced by stem-like cell characteristics. This mechanism may be responsible for acquiring resistance to Herceptin in the treatment of HER2-overexpressing breast tumors. Based on our findings, targeting pSTAT3 could overcome Herceptin-induced resistance in HER2-overexpressing breast tumors.

## Introduction

In breast cancer, HER2 proto-oncogene is notably overexpressed in about 25–30% and the patients and show poor prognosis, with a lower disease-free survival rate and a shorter time to relapse (1–6). HER2 has been proven to be an excellent target for therapy as HER2-driven mechanism has been linked to tumor growth, resistance to chemotherapy and metastasis (7,8). While Herceptin is being used as a first-line drug treatment for HER2-positive breast cancer, about 52% of breast cancer patients fail to respond to the initial Herceptin treatment or develop resistance to the antibody therapy within one year (9–11). Currently much effort is going into uncovering cellular factors that elicit the drug resistance in HER2-positive cancer. To date, the mechanism for this incurred resistance is poorly understood.

We have previously identified a specific upregulation of the transcription factor STAT3 from the HER2-overexpressing and ER-positive human breast cancer cell line MCF7-HER2 (unpublished data). The screen was performed with 44,000 genome-wide microarray of MCF7 cancer cells where STAT3 mRNA expression was increased by 3.62-fold in MCF7-HER2 cells compared with MCF7 wild-type. STAT3 is a latent cytoplasmic transcription factor that conveys signals from the cell surface to the nucleus by cytokines or growth factors (12). Clinically, increase in serum IL-6 levels has been correlated with a poor prognosis in breast cancer patients (13). STAT3 has been reported constitutively activated in about 50–60% of the primary breast tumors and activated through the phosphorylation of Y705 by cytoplasmic non-receptor tyrosine kinases (14–17).

Recent studies have revealed the potential roles of STAT3 in breast cancer. It was shown that STAT3-RANTES autocrine signaling is essential for tamoxifen resistance in breast cancer (18). Another group has demonstrated that the JAK2/STAT3 signaling was specifically required for the growth of CD44^+^/CD24^−^ stem cell-like breast cancer cells in human tumors (19). It was also reported that HER2 overexpression elicited IL6 secretion and activated STAT3, enforcing an autocrine loop of HER2-IL6-STAT3 expression (20). These data suggest that STAT3 signaling may play a crucial role for the chemotherapeutic resistance in human breast cancer.

In this work, we report that HER2 overexpression leads to activation of phosphorylation of STAT3 with subsequent upregulation of stem cell markers that may eventually lead to resistance to Herceptin treatment. Using HER2/ER-positive breast cancer model, we have demonstrated the reduction in the epithelial characteristics and subsequent increase in stem cell-like characteristics. Testing combined treatments of STAT3 inhibitor and Herceptin resulted in a significant inhibition of growth for HER2-overexpressing cancer cells. More importantly, HER2/ER/STAT3 activation signaling held true in human breast cancer patient tissues. Our results suggest that a combined treatment of STAT3 inhibitor with Herceptin may help overcome the incurred resistance derived from the enriched cancer stem-like cells in breast tumors. Considering that breast tumors co-express HER2 and ER, these results have important implications for targeted therapy.

## Materials and methods

### Breast cancer cell lines

BT474 wild-type, SKBR3 wild-type and MCF7 wild-type cells were obtained from the American Type Culture Collection (ATCC). They were maintained in a monolayer culture in DMEM/F12 (Dulbecco’s modified Eagle’s medium) with 10% fetal bovine serum, 2.5% L-glutamine and 0.5% penicillin/streptomycin. The MCF7-HER2 (MCF7 cells transfected with HER2) cell line was a generous gift of Dr C. Kent Osborne (Baylor College of Medicine, Houston, TX). MCF7-HER2 cells were maintained in a monolayer culture in DMEM 1X with 10% fetal bovine serum, 2.5% L-glutamine, 0.5% penicillin/streptomycin, and G418 (400 *μ*g/ml).

### Tumorsphere formation assay

Matrigel (BD, Cambridge, MA), 200 *μ*l was spread as a thick layer on a 24-well plate and allowed to polymerize at 37°C for 15 min, then 2×10^4^ cells grown as monolayer were trypsinized to single cells and plated on top of the pre-coated Matrigel. Plates were incubated at 37°C to allow cells to fully settle down before media was replaced with appropriate growth media containing 5% Matrigel. Cells were grown for 15 days; fresh growth media with Matrigel was replenished every two days. Images of representative fields were taken.

### Short hairpin interfering RNA transfection

The HER2 short hairpin RNA (shRNA) and control shRNA were purchased from the Ori Gene (Rockville, MD). The transfection was performed by using Lipofectamine 2000 (Invitrogen) reagent following the manufacturer’s instructions. The STAT3 shRNA was a kind gift of Dr Hyung-Gyoo Kang at the Children’s Hospital in Los Angeles. The HER2 and STAT3 shRNA knockdown expressions after shRNA transfection were determined by a Western blot analysis at 48–72 h of transfection.

### Western blot analyses

Monolayer cultures of respective cell lines at an 80–90% confluence were lysed using 100 *μ*l of RIPA (Thomas Scientific Co.) buffer. Tris-glycine (Bio-Rad) gels were loaded with 50–100 *μ*g of lysates. After running gel electrophoresis, the gel was transferred to a nitrocellulose membrane for 2 h. The membrane was blocked for 1 h in 5% BSA or 5% skim milk at 4°C. The membrane was then washed 3 times with 1X TTBS and incubated overnight with the primary antibody at 4°C. Primary antibodies of Oct-4, Sox-2, STAT3, pSTAT3, E-cadherin, vimentin, slug and β-actin were purchased from Cell Signaling Technology (Danvers, MA).

### Boyden chamber invasion assay

Mouse fibroblasts (NIH-3T3) were used as a chemo-attractant, and grown in a 24-well plate in 2 ml of the same media as MCF7-HER2 cells. MCF7 WT and MCF7-HER2 experimental cells were synchronized to an equal number (125,000 cells) in a 6-well plate and were serum starved overnight. Boyden chambers were prepared with 25 *μ*l of 1:6 diluted Matrigel and allowed to incubate for about 2 h to solidify. After cell synchronization, invasion was allowed to occur for 40 h. The cells were then fixed with 0.5% glutaraldehyde and stained with 5% toluidine blue for cell counting. Three different microscope fields of ×40 were used to quantify the invasion statistics when counting cells.

### FACS profile analysis

Approximately 500,000 cells of MCF7 WT or MCF7-HER2 cells were washed with 1X PBS, trypsinized and then transferred to a 15-ml aliquot tube. Cell suspensions were centrifuged down, re-suspended in 2 ml 1X PBS and then divided into two tubes 0.5 ml each for staining. One tube was used as an unstained control and the other one as stained with 10 *μ*l CD44 antibody (FITC Green; BD Biotech). The tubes were vortexed briefly and incubated at room temperature for 15 min in the dark. Each tube was then washed with 3.5 ml 1X PBS and then centrifuged for 6 min. After aspirating the supernatant, the cells were re-suspended in 3 ml 1X PBS and subjected to the FACS profiling at UCLA FACS Core Laboratory.

### Stattic (STAT3 inhibitor) treatment

All inhibitors were prepared as 200 mmol/l DMSO stocks for *in vitro* use. MCF7-HER2 cells that were at least 60–70% confluent were washed with 1X PBS and treated with 5 *μ*M of Stattic (STAT3 Inhibitor V; Santa Cruz Biotechnology) dissolved in 4 ml of 1X DMEM media. Untreated Stattic MCF7-HER2 cells were used as a control. Treatment lasted 24 h and cells were harvested for further analysis.

### MTT assay for dose response curve

MCF7-HER2 cells were cultured and counted and each test was conducted in triplicate. After mixing well, media/cell solution was diluted to 4,000 cells per 100 *μ*l. Cells were then plated in a 96-well plate: 6 wells for each treatment dosage, 4,000 cells per well (100 *μ*l). Cells were treated with Stattic (0 *μ*M control, 1, 5 and 10 *μ*M). Treatments lasted 48 h and then 50 *μ*l of MTT buffer reagent was added into each well. After incubation for 4 h at 37°C, 100 *μ*l of DMSO was added and gently agitated for 15 min. The plate was then read at 560 nm absorbance.

### Combined treatment of Stattic and Herceptin

The initial set up was similar to the one described in MTT assay dose response curve. This time, however, 10,000 cells were plated per 100 *μ*l in 8 wells per treatment. Cells were allowed to grow until about 50% confluence, and then treated with the following dosage treatments: untreated control, Stattic (5 *μ*M), Herceptin (10 *μ*g/ml, a gift of Genentech) and Stattic (5 *μ*M) + Herceptin (10 *μ*g/ml) combination. Treatments lasted 72 h and cell growth was monitored.

### Co-immunoprecipitation assay

To monitor the protein-protein interactions among HER2, ER and STAT3 proteins, ER specific monoclonal antibody (Santa Cruz Biotechnology, sc-7207) was added to the cancer cell lysates and incubated overnight. The immune-pellets were spun at 3,000 rpm for 5 min and washed three times with RIPA buffer (Thomas Scientific Co). The pellets were then ran on the PAGE gel and immunoblotted for HER2 and STAT3 proteins, respectively.

### Tissue microarray analyses

Human breast cancer tissue micro-array slides (BRC961) were purchased from US Biomax Inc. (Ijamsville, MD, USA). Breast cancer tissue microarray slides were stained using standard IHC methods with an IHC-validated phospho-specific STAT3 antibody (Tyr705; Cell Signaling Technology, D3A7).

### Statistics

For determining statistical significance in all quantifications, Student’s t-test was used; the data are presented as the mean ± SD, and considered significant at p<0.05.

## Results

### Activation of STAT3 in the HER2-overexpressing and ER-positive breast cancer model

**Figure 1.**
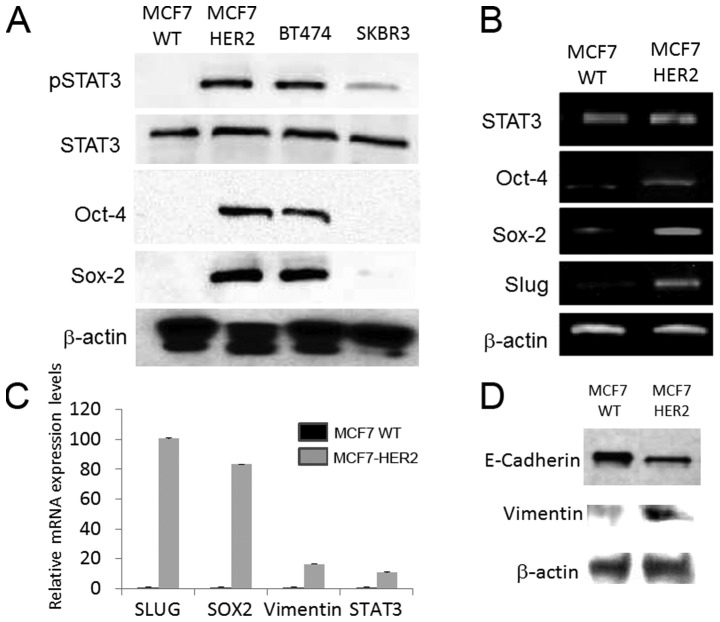
HER2 overexpression induced pSTAT3 and stem cell marker expression in ER-dependent manner. (A) Western blot analyses revealed that HER2 overexpression induced pSTAT3 and stem cell marker expression in MCF7-HER2. (B) RT-PCR confirmed that HER2 overexpression upregulated stem cell markers of Oct-4, Sox-2 and EMT driver slug in MCF7-HER2. (C) qPCR data showed the upregulation of CD44, vimentin, Oct-4 and Sox-2 in MCF7-HER2 cell line. (D) Western blot analyses with down-regulation of epithelial marker E-cadherin and upregulation of mesenchymal marker vimentin in MCF7-HER2.

Initially we tested the effect of STAT3 in HER2-positive breast cancer. We wished to determine whether the co-expression of HER2 and ER induced STAT3 phosphorylation, and whether pSTAT3 promotes stem-like cell phenotype in the breast cancer model. To this end, the basal expression of STAT3 and stem cell markers were examined in various human breast cancer cell lines by western blot analysis and real-time PCR. STAT3 was phosphorylated in MCF7-HER2 cells, but not in MCF7 wild-type (Fig. 1A). In addition, STAT3 was phosphorylated in BT474 as well as at very low level in SKBR3. MCF7 wild-type lacks HER2 amplification and SKBR3 lacks ER. This is consistent with previous data in which MCF7 WT did not typically show phosphorylation of STAT3 (21,22). Moreover, we found that the stem cell markers, Oct-4 and Sox-2, were expressed in MCF7-HER2 and BT474 cells, but not in MCF7 wild-type and SKBR3 (Fig. 1A). Real-time PCR analyses confirmed the upregulation of stem cell markers in HER2-overexpressing, ER-positive cancer cells (Fig. 1B). Our results support the hypothesis that HER2 over-expression and ER positivity promote STAT3 phosphorylation and induces the stem cell-like phenotype.

Transcription factor slug, which has been implicated as a driver for the epithelial-mesenchymal transition (EMT) was upregulated in MCF7-HER2 cells (Fig. 1B), leading us to the hypothesis that, HER2 induced stem cell marker expression and slug upregulation promoted the EMT phenotype in MCF7 cells. Real-time PCR analyses showed the gene expression pattern matching with the mesenchymal microenvironment, showing significant upregulation in vimentin, slug and concurrent downregulation of E-cadherin in MCF7-HER2 compared with control MCF7 wild-type (Fig. 1C). In line with the PCR data, western blot analyses revealed that epithelial marker E-cadherin was downregulated, while mesenchymal maker vimentin was upregulated in HER2 transfected MCF7 cells (Fig. 1D). These results indicate that the sequential activations elicited by HER2 amplification converge into the HER2-pSTAT3-stem cell markers - early EMT characteristics in signal pathways.

### STAT3 inhibitor abolished both the stem cell marker and EMT marker expressions

To uncover Herceptin resistance mechanism in HER2-overexpressing cancer cells, HER2/ER-positive cancer cells were treated with STAT3 inhibitor and examined for cancer stem cell phenotype. Stattic is a non-peptidic small molecule that inhibits STAT3 activation by selectively inhibiting dimerization and nuclear translocation of STAT3 (23). For the purpose of proving the effect of Stattic on inhibiting STAT3 and to investigate its effects on the HER2-ER-STAT3, we treated cells with 5 *μ*M Stattic for 24 h and monitored its effects on EMT and stem cell marker expression.

**Figure 2.**
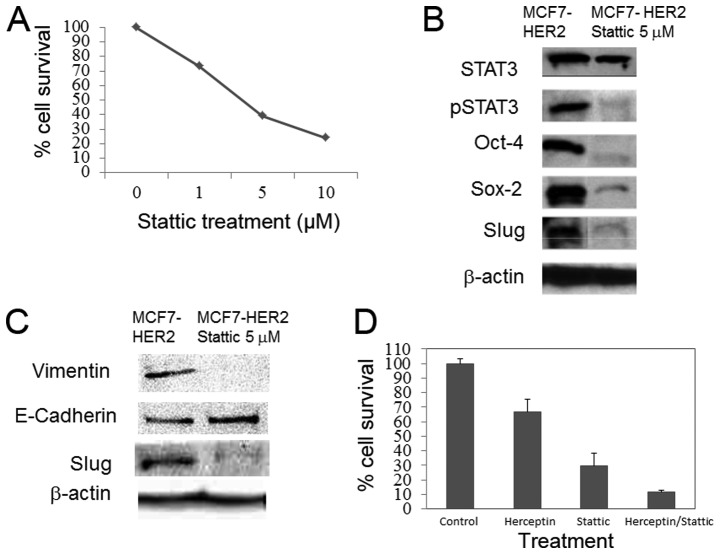
STAT3 inhibitor, Stattic abolishes the stem cell marker expression. (A) Dose response curve of MCF7-HER2 for the STAT3 inhibitor Stattic. (B) Stattic treatment abolished pSTAT3 and the stem cell marker expression in MCF7-HER2. The EMT driver slug expression was also abolished upon Stattic treatment. (C) Western blot analyses showed that, with Stattic treatment, expression of vimentin and slug was downregulated while E-cadherin expression was upregulated in MCF7-HER2. (D) Combined treatment of Herceptin and Stattic showed a synergistic cell growth inhibition effect on HER2-overexpressing, ER-positive breast cancer cells.

To begin with, dose response to Stattic was monitored in MCF7-HER2 cells (Fig. 2A). Stattic treatment of 5 *μ*M resulted in 28% of cell survival. Western blots of the MCF7-HER2 cells treated with 5 *μ*M of Stattic illustrated inhibition of STAT3 activation, as expected, but also showed significantly diminished Sox-2, Oct-4, and slug expression to the point of abrogation (Fig. 2B). While total STAT3 was almost intact with the Stattic treatment, pSTAT3 expression was also abolished simultaneously. For EMT marker expression, we monitored expression of E-cadherin, vimentin and slug protein. In agreement with cancer stem cell markers, the expression levels of EMT markers, vimentin and slug, were clearly decreased upon Stattic treatment (Fig. 2C). Our data suggest that STAT3 activation is responsible for the stem cell marker expression in HER2-overexpressing breast cancer cells.

### Combined treatment of Stattic and Herceptin show synergistic anti-growth effects

Given Stattic’s effect on reducing stem cell-like characteristics, we designed a combined treatment regimen consisting of Stattic, STAT3 inhibitor, and Herceptin, HER2 monoclonal antibody. The goal was to demonstrate that HER2-positive, ER-positive cancer cells were more sensitive to Herceptin treatment when also exposed to Stattic, due to the inhibition of STAT3. This would also help prove the presence of cross-talk between HER2/ER-STAT3 pathways through the synergistic effect of the combined treatment. Thus, we hypothesized that Stattic could help delay or even reverse incurred resistance associated with HER2 overexpression.

Based upon the dose response curve, it was estimated that 5 *μ*M would be the optimal dosage for the combined treatment. In a second MTT to determine the effectiveness of Stattic and Herceptin combined treatment, a control group, a solitary 10 *μ*g/ml Herceptin treatment group, a solitary 5 *μ*M Stattic treatment group and a combined 10 *μ*g/ml Herceptin with 5 *μ*M Stattic treatment group were compared to determine which treatment would provide the highest apoptosis rate. It was observed that while all treatments lead to significant reduction in the cell population based upon the control population, the Herceptin and Stattic combination treatment showed the highest rate of apoptosis (Fig. 2D). The combination treatment displayed significant effect (cell survival 11%) when compared to the solitary Herceptin and Stattic treatments.

### Targeted knockdown of STAT3 and HER2 expression significantly reduces the CD44^+^ subcellular population

**Figure 3.**
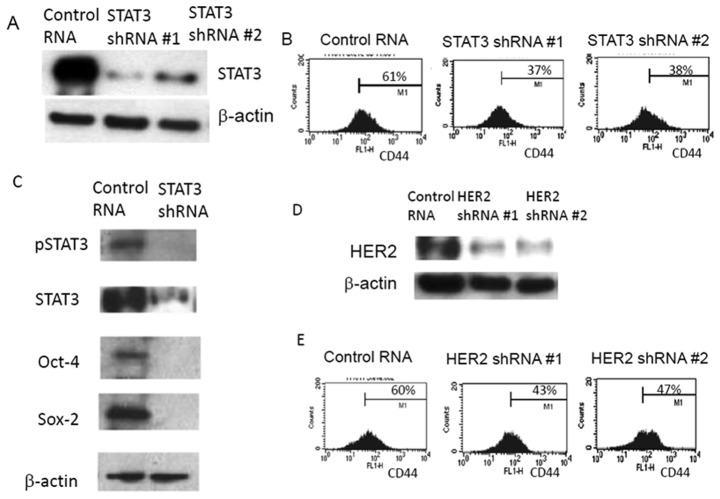
Targeted knockdown of STAT3 gene reduces CD44-positive cell populations. (A) shRNA driven targeted knockdown of STAT3 gene was performed with MCF7-HER2. STAT3 knockdown was confirmed with western blot analyses. (B) FACS profiling of CD44-positive sub-population from the control RNA and shRNA of STAT3 in MCF7-HER2 cells are presented. (C) Western blot analyses reaffirmed the stem cell marker abolishment upon STAT3 knockdown. (D) shRNA knockdown for HER2 gene was performed. Western blot analyses confirmed the HER2 gene knockdown. (E) FACS profiles of CD44-positive sub-populations from the control RNA and shRNA of HER2 transfected cancer cells.

STAT3 specific inhibitor Stattic treatment convincingly abolished the stem cell phenotype. To test whether STAT3 drives the stem cell phenotype in breast cancer cells, we knocked down STAT3 gene by shRNAs and examined the CD44^+^ subpopulation by fluorescence activated cell sorting (FACS). Targeted STAT3 knockdown was confirmed by western blot analysis and subsequently applied to FACS analysis (Fig. 3A). When STAT3 expression was knocked down with two independent shRNAs, the CD44(+) subpopulation has clearly reduced from 61.21% (CD44^+^ of the control RNA) to 37.22% and 38.23%, respectively (Fig. 3B). In agreement with FACS data, western blot analyses of STAT3 knockdown cells also showed downregulation of stem cell marker expressions (Fig. 3C). These results suggest that STAT3 plays a clear role in inducing the expression of stem cell markers Oct-4, Sox-2 and CD44.

Not only the expression level of stem cell markers of Oct-4 and Sox-2 was increased, but CD44(+) cell population was also increased in HER2-overexpressing, ER-positive breast cancer cells. To establish the potential link between HER2 amplifi cation and the stem cell phenotype, we knocked down the HER2 gene by shRNAs and examined CD44(+) FACS profile (Fig. 3D). The CD44(+) subcellular population was reduced from 60.77% to a range of 43.17-47.16%, when HER2 expression was knocked down by two independent HER2 shRNAs (Fig. 3E). This confirms that HER2 is also a driver for induction of the cancer stem cell-like phenotype in MCF7 cells.

### HER2 overexpression causes the increased cell invasiveness of breast cancer cells

**Figure 4.**
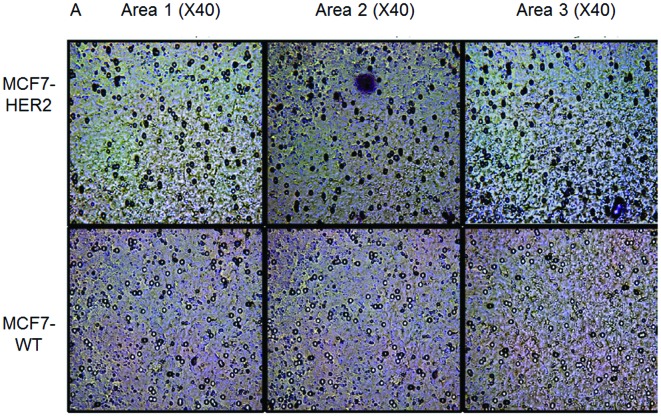

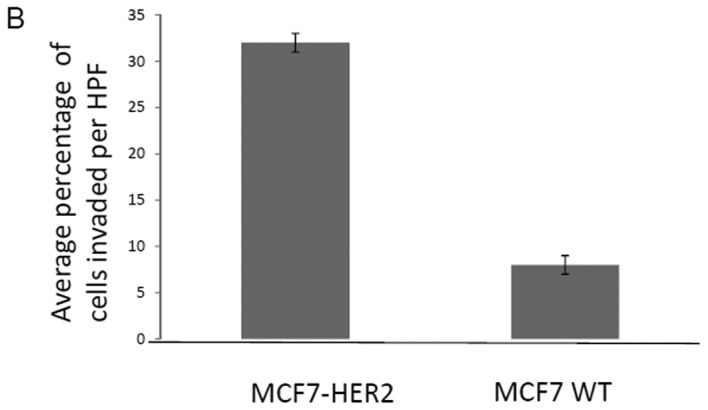
HER2 overexpressed cells display enhanced cell invasiveness *in vitro*. To measure the cell invasiveness of MCF7 WT and MCF7-HER2, cells were subjected to Boyden chamber assay. After 72 h of incubation, invaded cells were monitored and counted in 3 independent areas. (A) Results of Boyden chamber invasion assay was organized into three different microscopic fields (×40) depicting invaded MCF7 cells. Bluish-black cells by Toluidine blue indicate that the cell has invaded into the matrigel. (B) Graphic representation showing increased cell invasiveness in MCF7-HER2 cells compared to MCF7 WT cells based on the average number of cells invaded per high powered field (HPF). MCF7 WT averaged 8.3% invaded per HPF, while MCF7-HER2 averaged 31.3% invaded cells (p<0.05).

Cancer stem cell characteristics are associated with enhanced cell invasiveness. To determine whether forced HER2 overexpression leads to STAT3 activation and functionally enhances the invasiveness of MCF7 cells, an *in vitro* Boyden chamber invasion assay was implemented. As expected, MCF-7 wild-type control cells showed minimal invasion (8% of invasion population) through the Matrigel, even in the presence of NIH3T3 mouse fibroblasts as a chemoattractant (Fig. 4A). In contrast, MCF7-HER2 cells were 4 times more invasive (31%, Fig. 4B), as they averaged 23 more invaded cells per high powered field (HPF, ×40). Although MCF7 is typically known for being a non-invasive cell line, HER2 overexpression leading to STAT3 activation resulted in an increase of the invasiveness of the cell line.

### HER2 overexpression promotes tumorsphere formation whereas knockdown of STAT3 abolishes the sphere formation

**Figure 5.**
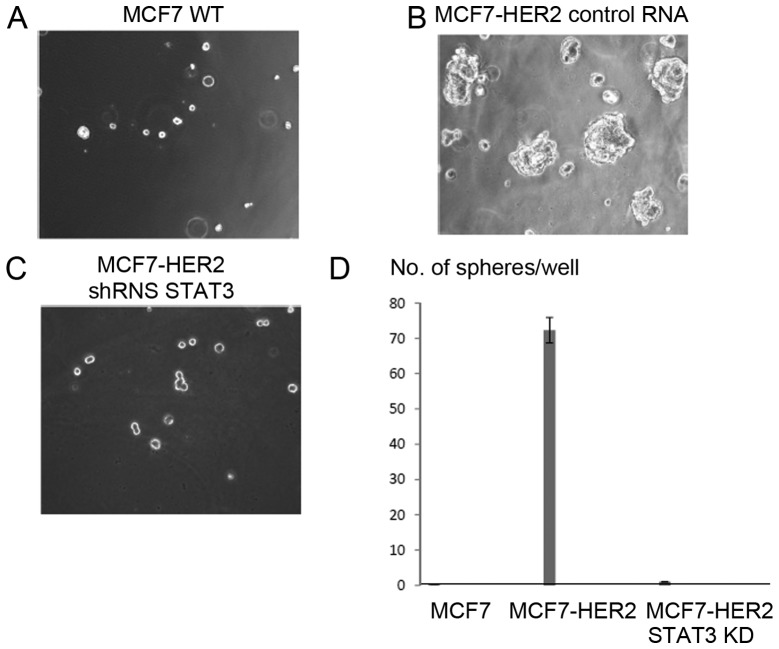
Tumorsphere formation in HER2-overexpressing cancer cells in 3D culture system. (A) MCF7 WT cells did not form any tumorspheres while MCF7-HER2 formed tumor spheres stably after day 6 (B). Targeted knockdown of STAT3 cells failed to form any spheres (C). The formed spheres were counted from each well and presented as a graph (D).

Formation of spheres by tumor cells is often directly correlated with cancer stem cell phenotype. Having considered Oct-4, Sox-2 and CD44 expression data, we wished to determine the tumorsphere formation capacity with HER2-overexpressing breast cancer. Approximately 20,000 cells were seeded on each Matrigel 3D well and tumorsphere formation was observed up to 15 days. While MCF7 WT did not form any spheres, HER2-overexpressing MCF7 cells formed stable tumorspheres at day 6 (Fig. 5A and B). To verify that the tumorsphere forming capacity is due to STAT3, we repeated the 3D culture with the targeted STAT3 knockdown cells. As shown in Fig. 5C, the figure, STAT3 knockdown cells did not form any spheres. The tumorspheres were counted for each well and presented as a graph (Fig. 5D). These results are consistent with the STAT3 knockdown effects on expression of stem cell markers Oct-4, Sox-2 and CD44. STAT3 is necessary for tumorsphere formation in HER2-overexpressing cancer.

### Phosphorylated STAT3 is identified in HER2-overexpressing, ER-positive human breast cancer tissues

**Figure 6.**
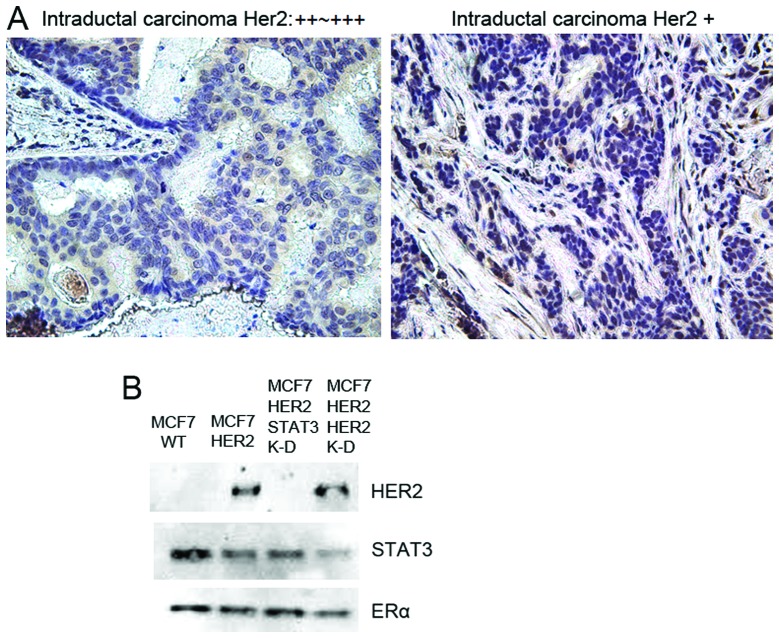
STAT3 activation in HER2-overexpressing, ER-positive human breast cancer. (A) Tissue microarray of human breast cancer patients. Breast cancer patient tissue microarray slides (BRC961) were obtained and stained using standard immunohistochemistry method with an IHC validated phospho-STAT3 antibody (Y705). pSTAT3-positive patient tissues are shown. Intraductal carcinoma HER2^++^-HER2^+++^, invasive ductal carcinoma HER2^+^. (B) Immunoprecipitation revealed the interactions between HER2/ER and STAT3. To examine the physical binding of ER, immunoprecipitation was performed with ERα antibody in MCF7-HER2 cells. For MCF7-HER2 cells, STAT3 K-D is STAT3 knockdown and HER2 K-D is HER2 knockdown cells. After IP, pellets were resolved on a PAGE gel and immunoblotted for HER2, STAT3 and ER proteins.

Recently, we found that STAT3 was upregulated by 3.64-fold in MCF7 cells transfected with HER2 compared to a MCF7 wild-type (12). However, the relationship between STAT3 in primary tumors and HER2 overexpression was not described. Thus, we hypothesized that HER2-positive, ER-positive breast cancer cells expressed phosphorylated STAT3. To test this, we have examined tissue microarrays of 71 primary breast tumors with previously confirmed ER, PR and HER2 expressions. We observed the phosphorylated STAT3 expression from these breast tumors by immunohistochemistry. We found that pSTAT3 was expressed only in HER2(+) and ER(+) primary breast tumors (Fig. 6A). As shown in the figure, 9 out of 17 HER2^+^/ER^+^ tumors expressed pSTAT3 and 6 out of 9 HER2^++^ or HER2^+++^/ER^+^ tumors showed pSTAT3 expression (Table I), moreover, 2 out of 4 showed pSTAT3 in ER/PR/HER2 triple negative patient tissues. This pSTAT3 activation is possibly through other STAT3 activation signaling axis in triple negative breast tumors such as Src or non-RTK phosphorylation mechanisms.

We also determined whether there are physical connections between HER2, ER and STAT3. Our next hypo thesis was that ER is bound to both HER2 receptor and STAT3 to transduce the phosphorylation signals from HER2 to STAT3. We used the MCF7-HER2 cell line for our model system of HER2(+)/ER(+) breast cancer. We immunoprecipitated the cell lysates with antibody against ERα and subsequently immuno blotted for HER2 and STAT3 in MCF7 wild-type, MCF7-HER2, MCF7-HER2 STAT3 knockdown and MCF7-HER2 HER2 knockdown cells (Fig. 6B). ERα was bound to both HER2 and STAT3 simultaneously in MCF7-HER2 cancer cells. When STAT3 is knocked down, HER2 is still bound to ER, when HER2 is knocked down, ER is still bound to STAT3. The tissue microarray and immunoprecipitation data suggest, for the first time, the existence of HER2-ER-STAT3 signaling axis in HER2-positive breast cancers.

## Discussion

We demonstrated herein that HER2 overexpression plays an essential role in inducing STAT3 phosphorylation, which further leads to increased expression of stem cell markers in human breast cancer cells. In particular, we show that co-expression of HER2 and ER induces phosphorylation of STAT3 in human breast tumors. This first established a signal transduction pathway of HER2-ER-STAT3 in HER2-positive breast cancer. Moreover, we demonstrated that HER2-ER-STAT3 activation led to the enhanced stem cell marker and EMT marker expressions. In relation to the acquired stem cell phenotype, Kong *et al* have shown that the expression of Sox-2 and Oct-4 are important indicators for cancer progression to metastasis and drug resistance (24). This supports the notion that HER2/ER overexpression activates STAT3 which leads to an increase in cancer stem cell markers, causing overexpression of HER2 and cells become resistant to chemotherapy.

Upon treatment with Stattic, we observed a significant reduction in the stem cell marker expression. In addition, when we knocked down the STAT3 gene, CD44^+^ subpopulation was reduced, suggesting the pivotal role of STAT3 in the cancer stem cell transition in HER2 amplified environment. More importantly, we found that MCF7-HER2 cells that were treated with Stattic, were more sensitive to Herceptin than MCF7 cells that were only treated with Herceptin or Stattic. This is a reflection of previous studies indicating that inhibited STAT3 has been correlated with increased apoptosis in cancer cells, increased chemosensitivity, suppressed tumor growth, reduced invasiveness, and decreased angiogenesis (25–27). Although STAT3 may play a vital role in early embryogenesis, its presence in the vast majority of adult cells is largely expendable, thus make it an attractive target for certain cancer therapies (28,29).

Our study, as well as others, suggests that STAT3 plays a crucial role in metastasis and therapeutic resistance in solid tumors. For example, STAT3 has been considered a fundamental component of resistant tumor growth in breast cancer (12,13), head and neck squamous cell carcinoma (27) and lung cancer (30,31) due to induction of an invasive EMT-like phenotype. There have been numerous studies linking the EMT phenomenon to the expression of stem cell-like characteristics to the point where they seem to overlap (32–34). Thus, given that EMT is associated with lasting tumor aggressiveness, invasion, and angiogenesis, it is considered a prime suspect for driving cancer stem-like cells.

Most of the reports arguing for EMT and cancer stem cell correlation focus on the idea that an EMT phenotype drives a cancer stem cell microenvironment that is characterized as CD44^hi^/CD24^low^ in breast cancer, which is associated with therapeutic resistance, tumor invasion and poor prognosis (35,36). As we observed in the current study, HER2 overexpression leading to STAT3 activation resulted in upregulation of CD44 expression. Furthermore, a recent study by Oliveras-Ferraros *et al* concluded that a mesenchymal CD44^+^/CD24^−^ microenvironment in HER2 overexpressed breast cancer was linked to resistance to Herceptin treatment (20).

We conclude that HER2 overexpression in ER-positive breast cancer results in STAT3 activation, further causing stem cell-like characteristics and resistance to Herceptin. We have found a model for commonly incurred Herceptin resistance. After an extended period of time constitutively activated STAT3, from HER2 and ER expression, may induce more and more resistant stem cell-like characteristics. While we used the specific STAT3 activation inhibitor Stattic in our study, other STAT3 activation inhibitors and shRNA should be explored for further effectiveness with Herceptin treatment.

## Figures and Tables

**Table I.** Summarized pSTAT3 staining from the tissue microarray of human breast tumors (US biomax, BRC961).^a^

	Total no. of cancer tissues	HER2/ER IHC-positive tissue numbers	Nuclear pSTAT3 IHC-positive tissue numbers
HER2^+^/ER^+^	71	17 (23.9% of 71)	9 (52.9% of 17)
HER2^++^-HER2^+++^/ER^+^	71	9 (12.6% of 71)	6 (66.6% of 9)
HER2^+^-HER2^+++^/ER^−^	71	24 (33.8% of 71)	0 (0% of 24)
HER2^−^/ER^−^/PR^−^	71	7 (9.8% of 71)	2 (28.5% of 7)

a pSTAT3 was scored from the pSTAT3 stained tissues. Only the tissues that showed more than 10% nuclear staining for pSTAT3 were counted for positivity.
