# [Corrigendum] STAT3 activation in HER2-overexpressing breast cancer promotes epithelial-mesenchymal transition and cancer stem cell traits

**DOI:** 10.3892/ijo.2025.5775

**Published:** 2025-07-14

**Authors:** Seyung S. Chung, Nolan Giehl, Yanyuan Wu, Jaydutt V. Vadgama

Int J Oncol 44: 403-411, 2014; DOI: 10.3892/ijo.2013.2195

Following the publication of the above article, a pair of interested readers drew to the Editor's attention that certain of the western blotting data featured in Figs. 1A and 3A were strikingly similar to data that had appeared in a pair of articles published previously by the same research group. Subsequently, an independent investigation of the data in this paper on the part of the Editorial Office revealed that a pair of the panels showing the results of cell invasion assays in Fig. 4A on p. 405 for the MCF7-WT cells appeared to contain overlapping sections, such that data which were intended to show results from entirely different microscopic fields had apparently been derived from partly the same original field of view.

Upon investigating these matters with the authors, they were able to repeat the experiments concerned (in the case of Figs. 1 and 3). The revised versions of Figs. 1, 3 and 4, now featuring the replacement data for Figs. 1A and 3A and the two completely differentiated microscopic fields of view for Fig. 4, are shown on the next two pages. The authors regret that certain of the data featured in Figs. 1 and 3 of this article were erronoeusly re-used from a pair of their previous publications, and thank the Editor of *International Journal of Oncology* for granting them the opportunity to publish this corrigendum. All the authors agree with the publication of this corrigendum; furthermore, they apologize to the readership of the journal for any inconvenience caused.

## Figures and Tables

**Figure 1:**
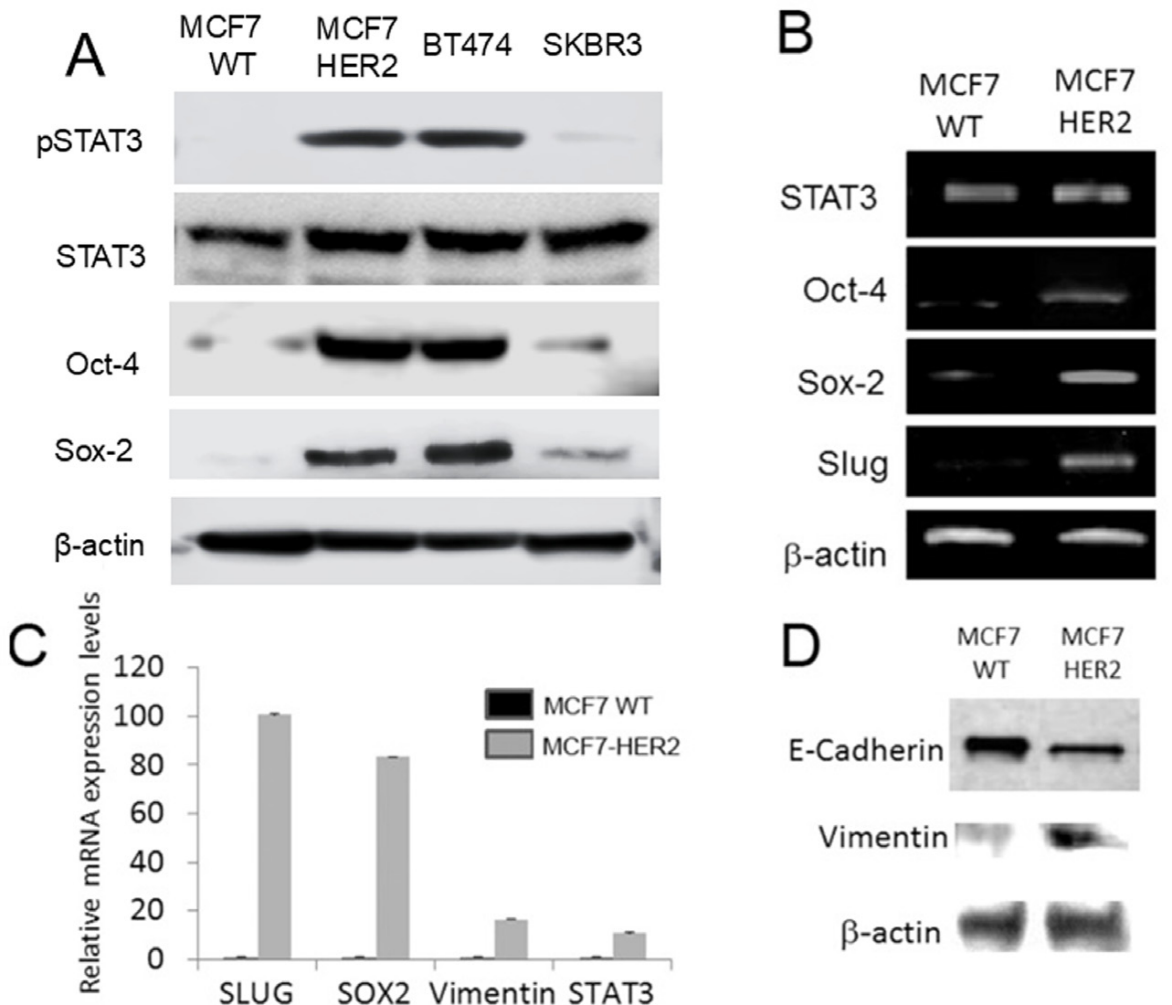
HER2 overexpression induced pSTAT3 and stem cell marker expression in ER-dependent manner. (A) Western blot analyses revealed that HER2 overexpression induced pSTAT3 and stem cell marker expression in MCF7-HER2. (B) RT-PCR confirmed that HER2 overexpression upregulated stem cell markers of Oct-4, Sox-2 and EMT driver slug in MCF7-HER2. (C) qPCR data showed the upregulation of CD44, vimentin, Oct-4 and Sox-2 in MCF7-HER2 cell line. (D) Western blot analyses with down-regulation of epithelial marker E-cadherin and upregulation of mesenchymal marker vimentin in MCF7-HER2.

**Figure 3:**
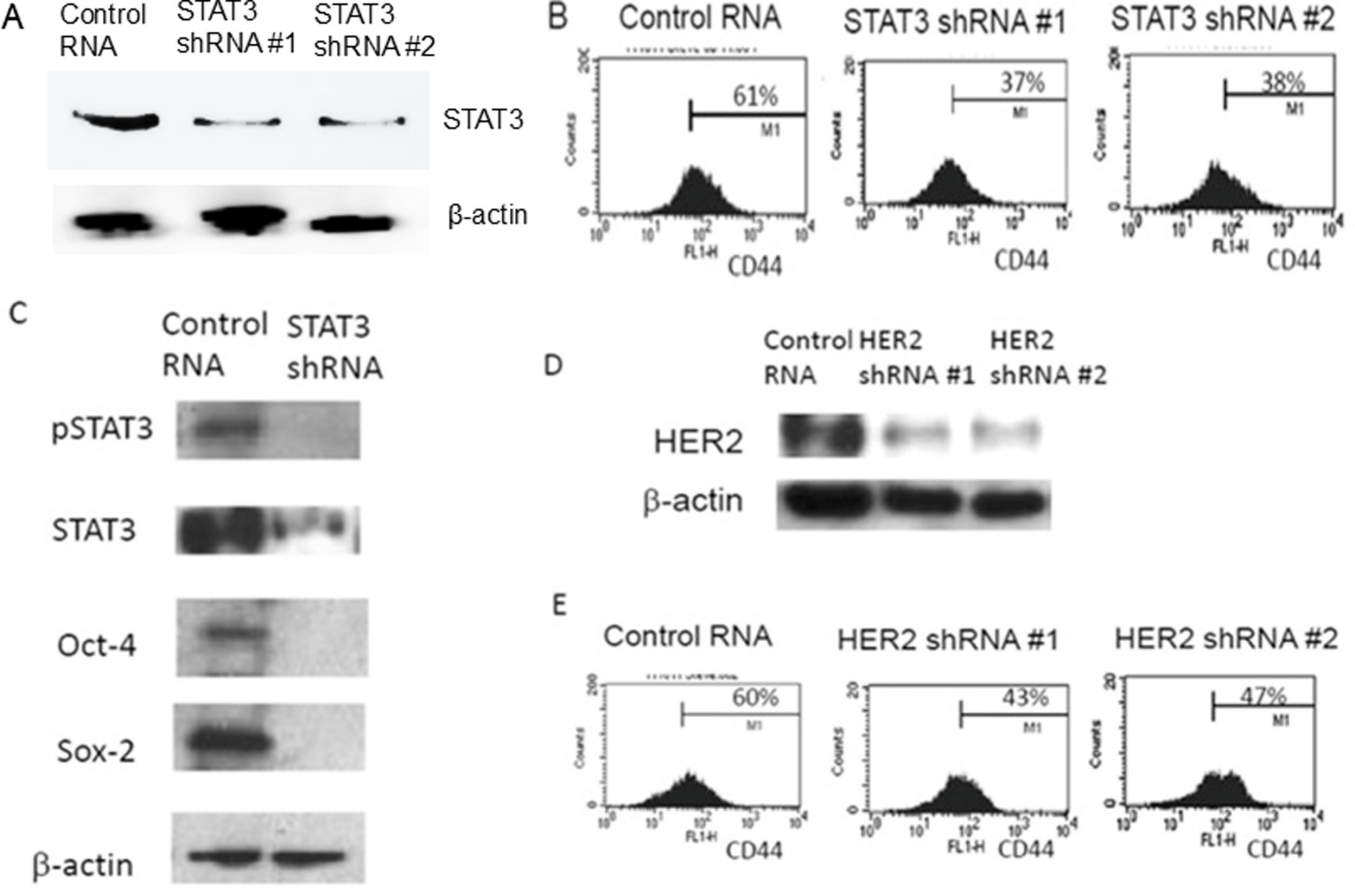
Targeted knockdown of STAT3 gene reduces CD44-positive cell populations. (A) shRNA driven targeted knockdown of STAT3 gene was performed with MCF7-HER2. STAT3 knockdown was confirmed with western blot analyses. (B) FACS profiling of CD44-positive sub-population from the control RNA and shRNA of STAT3 in MCF7-HER2 cells are presented. (C) Western blot analyses reaffirmed the stem cell marker abolishment upon STAT3 knockdown. (D) shRNA knockdown for HER2 gene was performed. Western blot analyses confirmed the HER2 gene knockdown. (E) FACS profiles of CD44-positive sub-populations from the control RNA and shRNA of HER2 transfected cancer cells.

**Figure 4:**
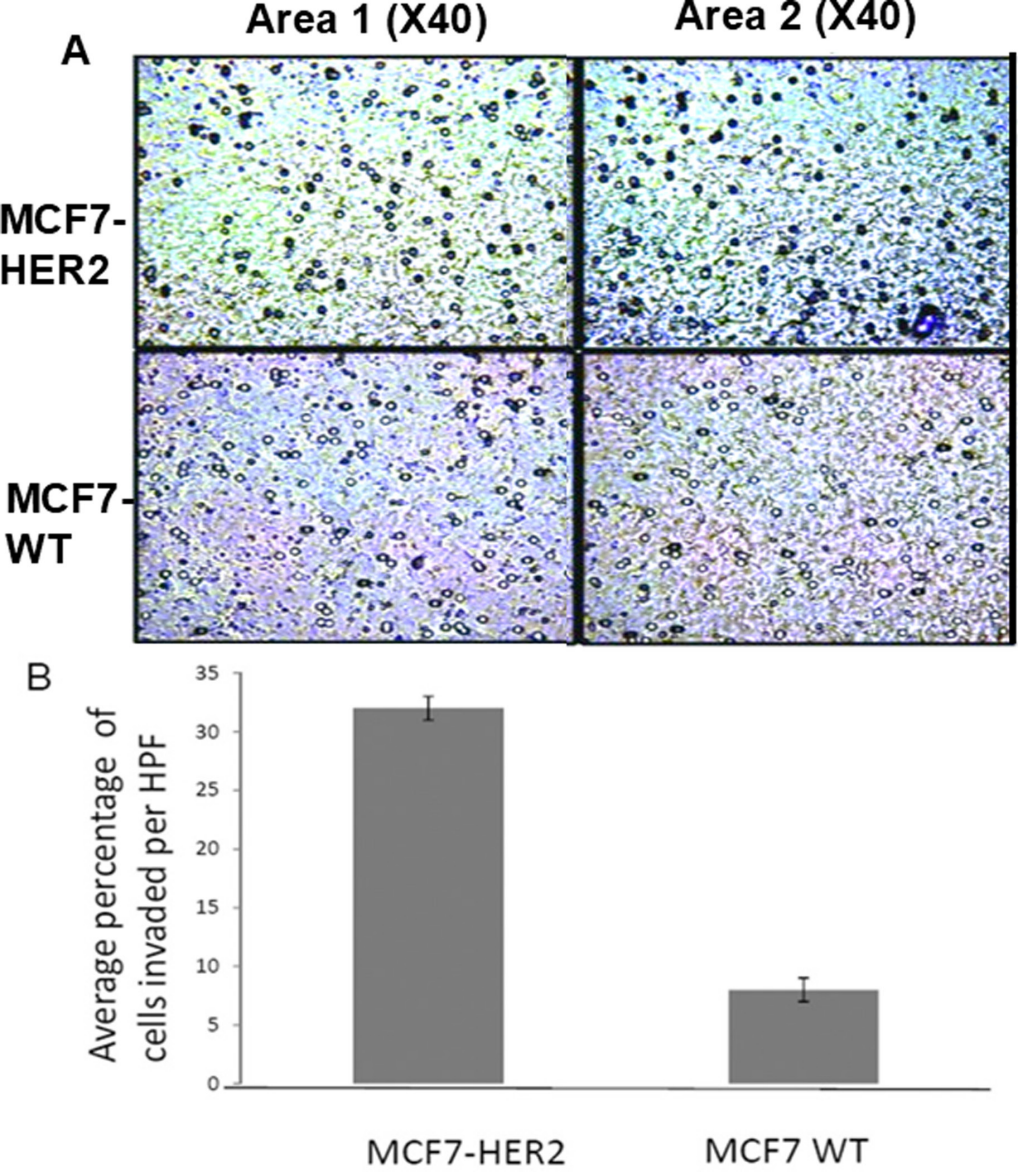
HER2 overexpressed cells display enhanced cell invasiveness *in vitro*. To measure the cell invasiveness of MCF7 WT and MCF7-HER2, cells were subjected to Boyden chamber assay. After 72 h of incubation, invaded cells were monitored and counted in 2 independent areas. (A) Results of Boyden chamber invasion assay was organized into two different microscopic fields (×40) depicting invaded MCF7 cells. Bluish-black cells by Toluidine blue indicate that the cell has invaded into the matrigel. (B) Graphic representation showing increased cell invasiveness in MCF7-HER2 cells compared to MCF7 WT cells based on the average number of cells invaded per high powered field (HPF). MCF7 WT averaged 8.3% invaded per HPF, while MCF7-HER2 averaged 31.3% invaded cells (p<0.05).

